# The Role of Histamine and Histamine Receptors in Mast Cell-Mediated Allergy and Inflammation: The Hunt for New Therapeutic Targets

**DOI:** 10.3389/fimmu.2018.01873

**Published:** 2018-08-13

**Authors:** Elden Berla Thangam, Ebenezer Angel Jemima, Himadri Singh, Mirza Saqib Baig, Mahejibin Khan, Clinton B. Mathias, Martin K. Church, Rohit Saluja

**Affiliations:** ^1^Department of Biotechnology, School of Bioengineering, SRM Institute of Science and Technology, Kattankulathur, Tamil Nadu, India; ^2^Department of Biochemistry, All India Institute of Medical Sciences, Bhopal, Madhya Pradesh, India; ^3^Discipline of Biosciences and Biomedical Engineering (BSBE), Indian Institute of Technology Indore (IITI), Indore, Madhya Pradesh, India; ^4^Central Food Technological Research Institute-Resource Centre, Lucknow, India; ^5^Department of Pharmaceutical and Administrative Sciences, Western New England University, Springfield, MA, United States; ^6^Department of Dermatology and Allergy, Charité – Universitätsmedizin Berlin, Berlin, Germany; ^7^Department of Biotechnology, Government of India, New Delhi, India

**Keywords:** histamine, histamine receptors, mast cells, allergy, inflammation, antihistamines

## Abstract

Histamine and its receptors (H1R–H4R) play a crucial and significant role in the development of various allergic diseases. Mast cells are multifunctional bone marrow-derived tissue-dwelling cells that are the major producer of histamine in the body. H1R are expressed in many cells, including mast cells, and are involved in Type 1 hypersensitivity reactions. H2R are involved in Th1 lymphocyte cytokine production. H3R are mainly involved in blood–brain barrier function. H4R are highly expressed on mast cells where their stimulation exacerbates histamine and cytokine generation. Both H1R and H4R have important roles in the progression and modulation of histamine-mediated allergic diseases. Antihistamines that target H1R alone are not entirely effective in the treatment of acute pruritus, atopic dermatitis, allergic asthma, and other allergic diseases. However, antagonists that target H4R have shown promising effects in preclinical and clinical studies in the treatment of several allergic diseases. In the present review, we examine the accumulating evidence suggesting novel therapeutic approaches that explore both H1R and H4R as therapeutic targets for histamine-mediated allergic diseases.

## Introduction

Allergic diseases, for example, allergic asthma, pruritus, atopic dermatitis, and allergic rhinitis are due to a complex interaction between several inflammatory cells, including basophils, mast cells, lymphocytes, dendritic cells, neutrophils, and eosinophils in response to various environmental/allergic stimuli (1, 2). These cells produce a plethora of inflammatory mediators, such as histamine, eicosanoids, chemokines, cytokines, and reactive oxygen species (3, 4). Among these, mast cell histamine is an axial player in stimulating the development of allergic-related inflammatory diseases by regulating the maturation and activation of leukocytes and directing their migration to target sites where they cause chronic inflammation (5–8). Histamine also exerts a various other immune regulatory functions by modulating the functions of monocytes (9), T cells (10, 11), macrophages (12), neutrophils (13), eosinophils (14), B cells, and dendritic cells (15). The biological impact of histamine follow their interaction with four types histamine receptors, H1R, H2R, H3R, and H4R, all of which belong to the G protein coupled receptor family (8, 16–20).

In this review, we focus on the importance and present knowledge about the histamine and histamine receptor-mediated activation in mast cell-mediated allergic disorders.

### Mast Cells: Source of Histamine

Mast cells are the major producer of histamine and express a vast array of receptors on their surface such as FcεR1, FcγRI, and receptors for complement components (C3aR and C5aR), nerve growth factor (NGF) (Trk A), substance P, vasoactive intestinal peptide (MrgX2), adenosine phosphate, etc. (21–24). Activation through these receptors by their respective stimulants, such as allergens, complement peptides C3a, C5a (25, 26), NGF (27), neuropeptides, adenosine mono-phosphate activate human cord blood-derived mast cells to release various inflammatory mediators including histamine. Histamine can also be produced by basophils and other immune cells (28) but much higher concentrations of histamine may be found in intestinal mucosa, skin, and bronchial tissues. Histamine regulates a plethora of pathophysiological and physiological processes, such as secretion of gastric acid, inflammation, and the regulation of vasodilatation and bronchoconstriction (29, 30). In addition, it can also serve as a neurotransmitter (31).

### Role of Histamine in Allergic Disease

Histamine plays a central role in the pathogenesis of several allergic diseases, such as atopic dermatitis, allergic rhinitis, and allergic asthma through differential regulation of T helper lymphocytes. Enhancement of Th2 cytokine secretion [such as interleukin (IL)-5, IL-4, IL-10, and IL-13] and inhibition of Th1 cytokine production [interferon-γ (IFN-γ), monokine IL-12, and IL-2] are mediated by histamine. Thereby, histamine regulates the effective balance between Th1 and Th2 cells by assisting a shift toward Th2 (32). Histamine-mediated mast cell activation plays a critical role in various allergic diseases. Histamine may induce the release of leukotrienes, cytokines, and chemokines *via* H4R in CD34^+^ cord blood-derived human mast cells (33). In mouse mast cells, both histamine and 4-methylhistamine can induce IL-6 production individually, an effect that is potentiated by LPS stimulation. This effect can be blocked by H4R antagonists and does not occur in H4R-deficient allergic mice (34). Recent findings have shown that activation of H4 receptors by histamine stimulates the synthesis of IL-4 and IL-5 in human cord blood mast cells and tumor necrosis factor (TNF)-α in bone marrow-derived murine mast cells (BMMCs), both of which have a potential role in inducing allergic inflammation (33, 35).

## Histamine Receptors and Their Role in Allergic Inflammation

Histamine receptors (H1R–H4R) are characterized by their function, structure, distribution, and their affinity to histamine (36, 37). Histamine has diverse effects, both pro-inflammatory and anti-inflammatory, which are determined by both the histamine receptor subtype and the cells stimulated types (38). The H1-receptor drives cellular migration, nociception, vasodilatation, and bronchoconstriction (39), whereas the H2-receptor modifies gastric acid secretion, airway mucus production, and vascular permeability (40). The H3-receptor plays an important role in neuro-inflammatory diseases (37). The H4-receptor has also been shown to be involved in allergy and inflammation (38, 41). H4R-mediated mast cell activation can regulate a powerful inflammatory cascade by releasing several inflammatory mediators; these mediators may stimulate the migration of different inflammatory cells into the inflammatory site (33). Likewise, the activation of H1R also regulates allergic responses by enhancing the migration of Th2 cells toward the allergen during lung inflammation (42). A more detailed summary of histamine receptor expression is shown in Table 1.

**Table 1:** Expression of different histamine receptors on various cells.

Histamine receptors	G-proteins	Expression on various cells	Reference
H1R	Gq/11	Human mast cells (skin, LAD2, intestinal mast cells)	(43–45)
		Tamm–Horsfall protein 1 (THP-1)	(33, 46)
		Nerve cells	(19)
		T-cells	(10, 11, 19)
		Airway and vascular smooth muscles	(19)
		Endothelial cells	(19)
		Epithelial cells	(47)
		Hepatocytes	(19)
		Chondrocytes	(19)
		Basophils	(19)
		B cells	(19)
		Peripheral blood mononuclear cell (PBMC)	(45)
		Eosinophils	(19)
		Neutrophils	(19)
		Dendritic cells	(15, 19)
		Natural killer cells	(15)
		SW756 cervical carcinoma cells	(44)
		Conjunctival fibroblast	(48)
		Macrophage	(12)

H2R	GαS	Human mast cells (skin, LAD2)	(43, 44)
		THP-1	(46)
		T-cells	(10, 11, 49)
		Conjunctival fibroblast	(48)
		Eosinophils	(50)
		PBMCs	(51, 52)
		Airway and vascular smooth muscles	(19)
		Endothelial cells	(19, 40)
		Epithelial cells	(19)
		Hepatocytes	(19)
		Chondrocytes	(19)
		Nerve cells	(19)
		B cells	(19)
		Monocytes	(19)
		Basophils	(19)
		Neutrophils	(53)
		SW756 cervical carcinoma cells	(44)

H3R	Gi/o	Neuroblastoma cell line MHH-NB-11	(45)
		Dendritic cells	(19)
		Eosinophils	(19)
		Histaminergic neurons	(19)
		Mast cells (LAD2)	(44)

H4R	Gi/os	Human mast cells (skin, LAD2, HMC-1, cord blood mast cells, intestinal mast cells)	(33, 43–45)
		Bone marrow and peripheral hematopoietic cells	(19)
		THP-1	(33)
		SW756 cervical carcinoma cells	(44)
		Nerve cells	(40)
		Conjunctival fibroblast	(48)
		PBMC	(45)
		T-cells	(19, 49, 54)
		Natural killer cells	(15)
		Basophils	(6, 55)
		Eosinophils	(5, 7, 50)
		Neutrophils	(19, 40)
		Monocytes-derived dendritic cells	(56)
		Macrophage	(40)
		Myeloid cells	(57)
		Dendritic cells	(15, 19)

### The H1-Receptor

**Figure 1:**
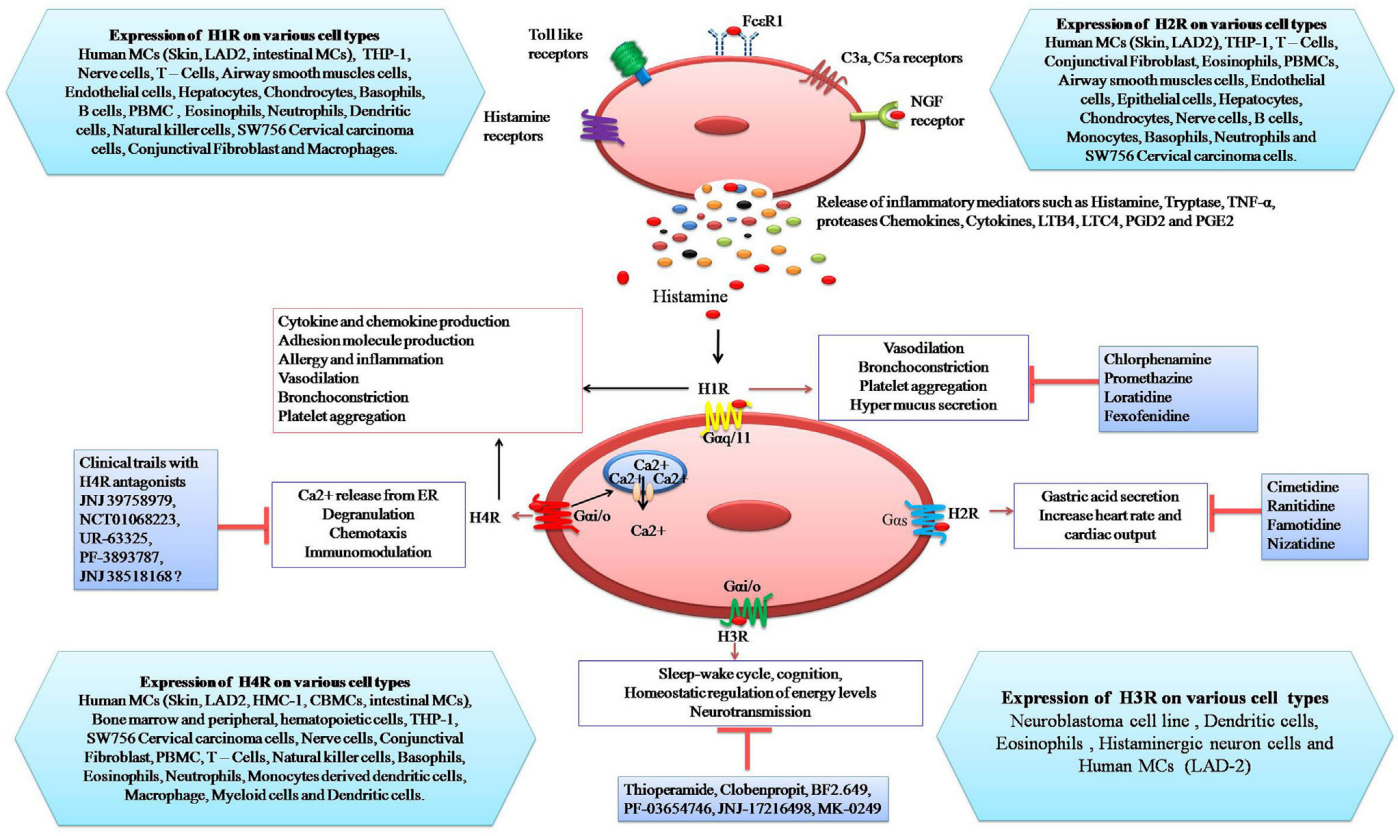
Schematic representation of the expression of histamine receptors on mast cells and their potential response to histamine: binding of histamine to H1R induces vasodilatation, bronchoconstriction, platelet aggregation, and mucus hyper-secretion. Stimulation of H2R by histamine causes gastric acid secretion, increase heart rate, and cardiac output. Activation of H3R is involved in sleep-wake cycle, cognition, homeostatic regulation of energy levels, and neurotransmission. H4R activation leads to Ca^++^ release from endoplasmic reticulum, degranulation, chemotaxis, and immuno-modulation whereas inhibitors of histamine receptors (H1R–H4R) inhibit specific responses.

The H1R is ubiquitously expressed and is involved in allergy and inflammation. H1R is expressed in many tissues and cells, including nerves, respiratory epithelium, endothelial cells, hepatic cells, vascular smooth muscle cells, dendritic cells, and lymphocytes (8, 19). Histamine activates H1R through Gαq/11, which then activates phospholipase C and increases intracellular Ca^++^ levels. As a consequence, histamine elicits the contraction of smooth muscle of the respiratory tract, increases vascular permeability, and induces the production of prostacyclin and platelet activating factor by activating H1R (Figure 1) (58). Thus, almost all immediate hypersensitivity reactions, including symptoms observed in the skin, such as erythema, pruritus, and edema, may be elicited by the activation of H1R (59).

Activation *via* H1R may also enhance both Th1- and Th2-type immune responses (11). In mice, deletion of H1R leads to the release of Th2 cytokines (IL-4 and IL-13) and inhibition of IFN-γ (60). Similarly, Bryce et al. (42) demonstrated that allergen-challenged H1R-deficient mice had attenuated lung allergic responses. They also demonstrated that histamine may act as a chemotactic factor for Th2 cells, stimulating their migration into lung tissues (42).

In addition, IL-3 activation can increase H1R expression on Th1 cells (61, 62), and histamine can enhance B cell proliferation, which is absent in H1R-deficient mice (63). The role of H1R activation in asthma may be further corroborated by observations showing that use of H1R-antagonists can significantly decrease asthma symptoms and improve pulmonary function in persistent asthma (58, 64, 65).

Histamine H1 receptor is also expressed in dermal dendritic cells and keratinocytes in the skin tissue, and histamine increases the NGF production *via* H1R in human keratinocytes (66). The secretion of NGF is caused by the phosphorylation of protein kinase C, extracellular signal-regulated kinases (ERK), and the activation of AP-1 resulting from H1R stimulation. Similarly, histamine, acting *via* H1R, has also been shown to enhance the production of chemokines, such as granulocyte macrophage colony stimulating factor, regulated on activation T cell expressed and secreted (RANTES), and monocyte chemotactic protein-1 (MCP-1) in IFN-γ-stimulated keratinocytes. It also upregulates the antigen-presenting capability of dendritic cells, and leads to Th1 polarization through H1R (67).

Histamine induces IL-31 production, which plays an important and crucial role in pruritus and skin barrier function in allergic dermatitis (54). Administration of an H1R antagonist decreased IL-31 levels in the serum of atopic dermatitis patients (68). These data therefore suggest that H1R activation by histamine has the ability to induce various symptoms related with allergic skin diseases such as pruritus and atopic dermatitis.

### The H2-Receptor

The Gαs-coupled H2R is highly expressed in various cells and tissues, such as B cells, T cells, dendritic cells, gastricparietal cells, smooth muscle cells, and the brain and cardiac tissues (Table 1). Activation of the receptor can induce airway mucus production, vascular permeability, and secretion of gastric acid (69). The role of the H2R is well studied in histidine decarboxylase knockout mice (HDC^−/−^) models which suggest that the lack of histamine can enhance downregulation of H2R expression in a tissue-specific manner (70). Furthermore, the H2R is importantly accountable for the relaxation of the airways, uterus, and smooth muscle cells in the blood vessels. Moreover, the H2R is involved in the activation of the immune system, such as Th1 cytokine production, reduction of basophil degranulation, T-cell proliferation, and antibody synthesis (71, 72). Knockdown of H2R^−/−^ mice show impaired immune functions, gastric acid secretion, and cognitive function associated with hippocampal potentiation impairments (73, 74) and nociception abnormalities (75).

### The H3-Receptor

The H3R is coupled to Gαi/o and exclusively expressed in neurones. It is important for homeostatic regulation of energy levels, sleep-wake cycle, cognition, and inflammation (76) (Figure 1). H3R-deficient mice exhibit altered behavior and locomotion (77) and display a metabolic syndrome characterized by obesity, hyperphagia, and increased leptin and insulin levels (78, 79). Similarly, several studies suggest that H3R knockout can also lead to an increase in severity of neuro-inflammatory diseases and can enhance the expression of IFN-inducible protein 10, MIP 2, and CXCR3 in T cells (80). These investigators also showed that H3R can be involved in blood–brain barrier function.

The H3R has also been associated with rhinitis (81). This is likely because it is expressed on presynaptic nerves in the peripheral sympathetic adrenergic system and also on nasal sub-mucosal glands. Stimulation of H3R suppressed norepinephrine release at presynaptic nerve endings and stimulated nasal sub-mucosal gland secretion (82).

Currently, several H3R ligands are available, but not in clinical use. H3R antagonists, such as clobenpropit and thioperamide, were extensively used as a research tools and few early stage clinical trial reports are also available for H3R antagonists (83). However, these antagonists are used to treat obesity, myocardial ischemic arrhythmias, cognition disorders, and insomnia (84).

### The H4-Receptor

The histamine H4R is coupled to Gα/io proteins (85) and is expressed on a variety of immune cells as well as on other cells such as spleen, intestinal epithelia, lung, synovial tissue, central nervous system, sensory neurons, and cancer cells (86–94). Stimulation of H4R reduces forskolin-induced cyclic AMP formation, which leads to the activation of MAPK and enhanced Ca^++^ release (6, 95). H4R mediates the pro-inflammatory responses of histamine in both autocrine and paracrine manners. Histamine enhances adhesion molecule expression, cell shape change, and cytoskeletal rearrangement *via* H4R, leading to the increased migration of eosinophils (5).

In various allergic diseases allergen cross linkage of FcεRI is the primary driver of mast cell activation. However, H4R is constitutively expressed on human mast cells such as LAD-2 and HMC-1 (33, 43). H4R-mediated activation of mast cells leads to the expression of various pro-inflammatory cytokine and chemokines, such as IL-6, TNF-α, TGF-β1, RANTES, IL-8, MIP-1α, and MCP-1 (33). Histamine H4R stimulation of mast cells may have three positive effects. First, it increases chemotaxis of mast cells thus encouraging their accumulation at the site of an allergic response (6). Second, it upregulates the expression of FcεRI on mast cells, thereby priming them for allergen-induced activation (96). Third, it mobilizes intracellular calcium to either prime mast cells for activation or, indeed, induce degranulation. These effects have been studied using histamine, the H4R-agonist 4-methylhistamine, the H4R-antagonists thioperamide or JNJ 7777120 and mast cells from H4R-deficient mice.

Basophils also express H4R on their surface and release histamine following antigen stimulation (55). However, basophils and mast cells differ in several important aspects, such as anatomical localization, the production of cytokines, and antigen-presenting activity. Histamine, acting *via* H4R, induces chemotaxis of bone marrow-derived basophils. H4R may play significant roles in basophil regulation in allergic dermatitis (97).

Among the Th subsets, the mRNA and protein of H4R are preferentially expressed in Th2 cells over naive T cells and Th1 cells. H4R may be involved in the pathogenesis of allergy and inflammation by activating Th2 as well as Th17 cells (68, 98). In human Th17 cells, H4R antagonists inhibit the IL-17 production, induced by H4R agonist (68).

Stimulation of H4R can also enhance the migration of eosinophils and the recruitment of mast cells leading to the amplification of immune responses and chronic inflammation. Similarly, H4R are involved in T cell differentiation and dendritic cell activation and its immunomodulatory function (6). Histamine and selective H4R agonists were shown to induce the shape change of eosinophils, an effect that maybe blocked by selective H4R antagonists (5). Treatment with JNJ 39758979 (H4R antagonist) resulted in a statistically significant inhibition of eosinophil shape change. These results showed that administration of H4R antagonists may have an impact on eosinophil function (38).

Finally, the activation of H4R involves several signaling cascades for the release of various allergic inflammatory mediators. ERK is a member of MAPK family and mediates the proliferation, differentiation, anti-apoptosis, regulation, and cytokine expression at gene level. There are reports showing that histamine can induce phosphorylation of ERK through H4R in peripheral blood derived CD34^+^ human mast cells as well as in mouse BMMCs (34) and HEK-293 cells (99). H4R-involved ERK and PI3 kinase pathways have been shown to be involved in the release of IL-6 in mouse BMMCs (34) and JAK/STAT signaling pathways for the release of TNF-α in a rat model (100). Recent studies (101) demonstrated that the activation of NFκB through H4R has followed the JAK/STAT signaling pathway.

## H4R: A Novel Drug Target for Allergic Diseases

In addition to H1R, H4R is considered as a novel drug target for the treatment of allergy and inflammation. Recently, the H4R antagonists such as JNJ 7777120 and JNJ 39758979 have been extensively used as a tools to understand the pathophysiological involvement of H4R and have been studied extensively in both cell culture and *in vivo* animal models (102, 103). Furthermore, H4R antagonists have been used to explore the role of H4R in allergic inflammatory disorders, such as allergic asthma, allergic rhinitis, and chronic pruritus (31).

## Role of Antihistamines in Mast Cell-Associated Diseases

Mast cells play an active role in various allergic diseases such as acute pruritus, atopic dermatitis, allergic asthma, allergic rhinitis, and pulmonary fibrosis (104, 105). H_1_-antihistamines, such as azatadine, cetirizine, and mizolastine are used for the treatment mast cell activated diseases (106). Cimetidine, ranitidine, famotidine, and nizatidine are H2R selective antihistamines that reduce gastric acid secretion (107). H3R antihistamines include thioperamide, clobenpropit, BF2. 649, PF-03654746, JNJ-17216498, and MK 0249.

JNJ 7777120 is a selective H4R antihistamine that is widely used in inflammation and pruritus (108). There are some H4R antihistamines which are under clinical trial, such as JNJ 39758979, NCT 01068223, UK-63325, PF-3893787, and JNJ 38518168 (Figure 1) (108, 109). H_1_-antihistamines are a standard treatment for mast cell-mediated allergic diseases. There is increasing evidence that histamine binding to H4 receptors exacerbates allergy and inflammation. Indeed, mast cells themselves have H4 receptors which when stimulated increased degranulation and cytokine production. Therefore, antihistamines targeting both the H1 and H4 receptor could be an effective treatment for mast cell-mediated allergic diseases (110).

## Clinical Trials Targeting Histamine Receptors

Pharmacological properties of H4R have been exhibited by various H4R transfected cells (87, 89, 99, 111, 112). It was observed that H1R and H2R specific agonist/antagonists cannot bind to the H4R. However, some H3R ligands such as imetit, clobenpropit, thioperamide, and *R*-methylhistamine are also able to bind to the H4R with different affinities. Currently, a number of H4R antagonists have been developed but only a few are undergoing clinical trials. JNJ 39758979, a potent and selective H4R antagonist, has shown impressive results in different allergic inflammatory diseases such as dermatitis, asthma, pruritus, and arthritis (102, 103).

Recent clinical trials (NCT01068223) with the H4R antagonist JNJ 39758979 help to demonstrate a significant role of the H4 receptor in pruritus in humans. Interestingly, the combination therapy of this H4R antagonist and the H1R antihistamine, cetirizine, showed a more beneficial effect in the treatment of pruritus as compared with H1R alone (113–116). Furthermore, a study was carried out by using JNJ 39758979 to treat persistent asthma (NCT00946569), but no results have yet been reported. There are some H4R antagonists under the clinical trial including toreforant (JNJ 38518168), PF-3893787, and UR-63325. Toreforant (JNJ 38518168) has been used for the treatment of rheumatoid arthritis (clinical trial numbers: NCT01679951, NCT00941707, and NCT01862224). However, a study in rheumatoid arthritis (NCT01679951) was terminated due to issues related to efficacy. Even though, studies are still going on with the H4R antagonist toreforant (JNJ 38518168) in patients with asthma and psoriasis (clinical trial numbers NCT01823016 and NCT02295865, respectively) (38).

## Conclusion and Future Prospective

The recent developments in research on histamine pathway underscore the importance of histamine in allergic inflammation through its effects on the H1R and H4R. Although, drugs targeting H1R are being explored for the treatment of various mast cell-associated allergic disorders, they are not always clinically effective. Several H4R antagonists have entered the later stages of clinical trials for a different range of allergic and inflammatory diseases. However, their clinical efficacy reports are not yet published. Furthermore, there appears to be some overlap in function between H1R and H4R, opening up the possibility for using synergistic strategies for therapeutic approaches. As such, we suggest the combination therapies by using both H4R together with H1R antagonists may provide a potential benefit in the treatment of various allergic and inflammatory diseases.

## Author Contributions

ET, EJ, HS, MB, MK, CM, MC, and RS designed the manuscript; were involved in drafting/revising the manuscript; and read and approved the final manuscript. ET, EJ, and RS wrote the first draft.

## Conflict of Interest Statement

The authors declare that the research was conducted in the absence of any commercial or financial relationships that could be construed as a potential conflict of interest.

## Abbreviations

BMMCs, bone marrow-derived murine mast cells; ERK, extracellular signal-regulated kinases; GM-CSF, granulocyte macrophage colony stimulating factor; H1R, histamine H1 receptor; H2R, histamine H2 receptor; H3R, histamine H3 receptor; H4R, histamine H4 receptor; IFN-γ, interferon-γ; IL, interleukin; MCP-1, monocyte chemotactic protein-1; NGF, nerve growth factor; PKC, protein kinase C; TNF, tumor necrosis factor; RANTES, regulated on activation T cell expressed and secreted; PBMC, peripheral blood mononuclear cells; THP-1, Tamm–Horsfall protein 1.
